# Supplementary material for: Rationally patterned electrode of direct-current triboelectric nanogenerators for ultrahigh effective surface charge density
Source: Nat Commun. 2020 Dec 3;11:6186. doi: 10.1038/s41467-020-20045-y (PMC7712892; doi:10.1038/s41467-020-20045-y)
Supplement: Supplementary file 1 — Supplementary Information [file 41467_2020_20045_MOESM1_ESM.pdf]

Supplementary Materials

Rationally patterned electrode of direct-current triboelectric nanogenerators for ultrahigh effective surface charge density

Zhihao Zhao[1,2], Yejing Dai[2], Di Liu[1,3], Linglin Zhou[1,3], Shaoxin Li[1,3], Zhong Lin Wang[1,3,4,*] and Jie Wang[1,3,*]

[1]Beijing Institute of Nanoenergy and Nanosystems, Chinese Academy of Sciences, Beijing 100083, P. R. China

[2]School of Materials, Sun Yat-sen University, Guangzhou 510275, P. R. China

[3]College of Nanoscience and Technology, University of Chinese Academy of Sciences, Beijing 100049, P. R. China

[4]School of Materials Science and Engineering, Georgia Institute of Technology, Atlanta, GA 30332, USA

[δ]Z. Zhao, Y. Dai, and D. Liu contributed equally to this work.

[*]Corresponding Author: Z. L. Wang: zhong.wang@mse.gatech.edu; J. Wang: wangjie@binn.cas.cn

Content

Supplementary Figures:

Supplementary Fig. 1 Mechanism of DC-TENG.

Supplementary Fig. 2 Structure and output performance of DC-TENGs with different friction electrode lengths.

Supplementary Fig. 3 Photograph of MDC-TENG device with 20 units.

Supplementary Fig. 4 Short-circuit current of MDC-TENG under different sliding distances.

Supplementary Fig. 5 Output of DC-TENG (single electrostatic breakdown device) with gap distance from 40 μm to 550 μm.

Supplementary Fig. 6. Short-circuit current of MDC-TENG at various gap distances.

Supplementary Fig. 7. Output performance of MDC-TENG device with gap distance ~0.

Supplementary Fig. 8 Simulated electric field distribution in the gap of MDC-TENG.

Supplementary Fig. 9 Electric field distribution under different gap distance.

Supplementary Fig. 10 Open-circuit voltage of MDC-TENG with various MDC-TENG units.

Supplementary Fig. 11. Charge density of MDC-TENG with various friction layers.

Supplementary Fig. 12 Circuit of MDC-TENG driving electronic devices directly.

Supplementary Fig. 13 Circuit of self-powered MDC-TENG system.

Supplementary Fig. 14. The SEM images of PTFE film surface.

Supplementary Notes:

Supplementary Note 1: Working mechanism of DC-TENG.

Supplementary Note 2: Development of charge density improvement for AC-TENG.

Supplementary Note 3: Relationship between output charge and sliding distance.

Supplementary Note 4: Relationship between I_{sc} and velocity.

Supplementary Note 5: Relationship between dI/dt and acceleration.

Supplementary Note 6: Calculation of average strength of electrostatic field.

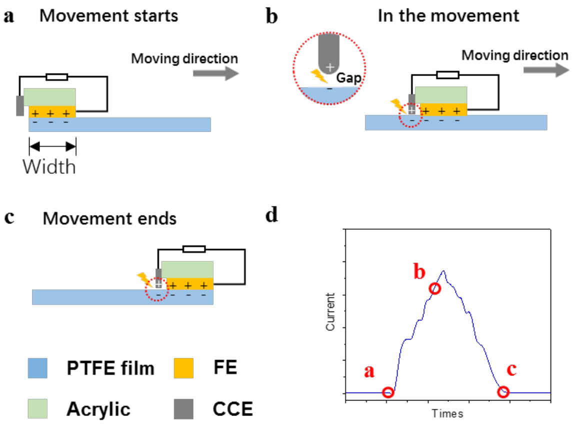

Supplementary Fig. 1. The mechanism of DC-TENG. (**a-c**) The schematic diagram of DC-TENG at different movement condition and (**d**) corresponding output current.

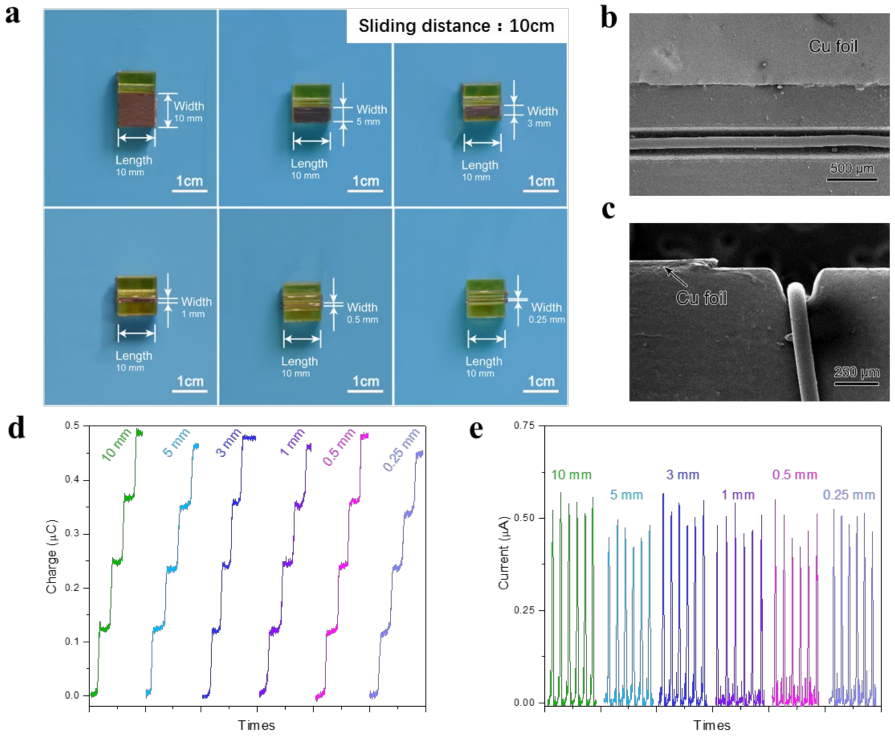

Supplementary Fig. 2. The effect of electrode width on the output performance of DC-TENG. (**a**) The photographs of DC-TENGs with different friction electrode widths (electrode length: 10 mm). (**b, c**) SEM images of the surface and cross section of DC-TENG. (**d**) The output charges and (**e**) short-circuit currents of DC-TENG with various friction electrode widths (electrode length: 10 mm, sliding distance: 10 cm).

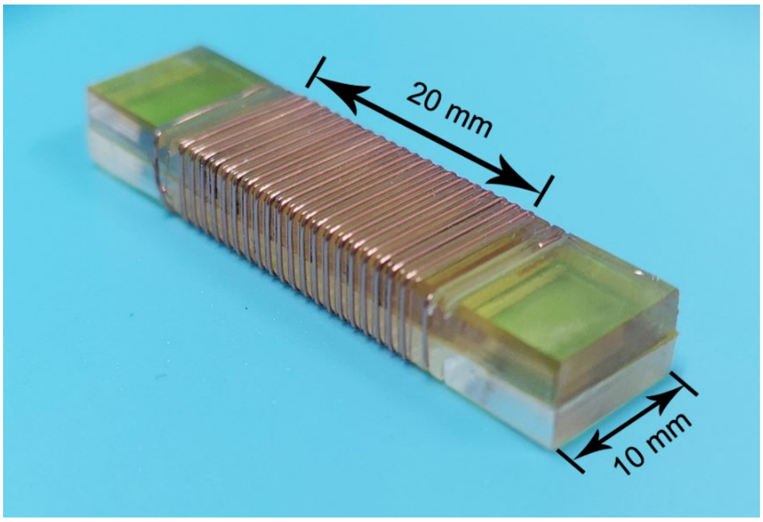

Supplementary Fig. 3. Photograph of MDC-TENG device with 20 units.

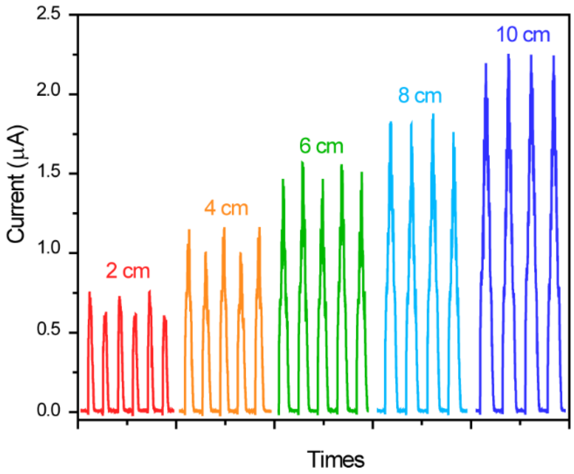

Supplementary Fig. 4. The I_{sc} of MDC-TENG under different sliding distances.

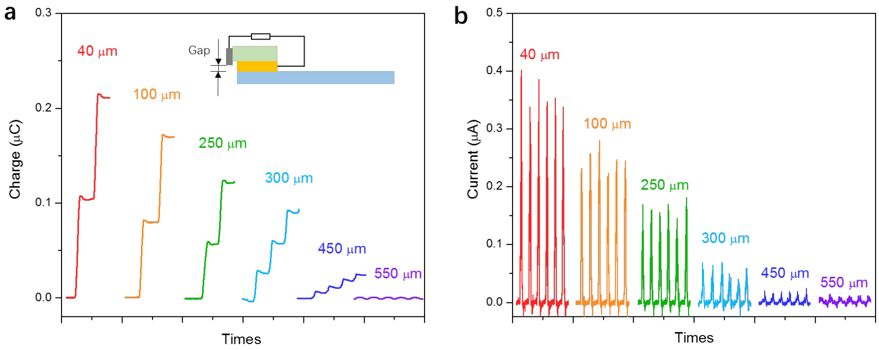

Supplementary Fig. 5 The output of DC-TENG (single electrostatic breakdown device) with gap distance from 40 μm to 550 μm: (**a**) output charge and (**b**) short circuit current.

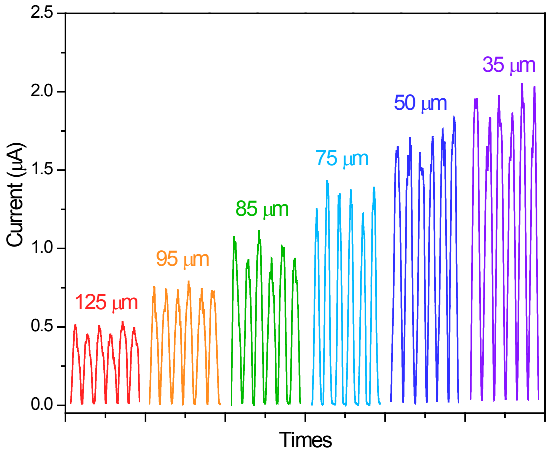

Supplementary Fig. 6. The short-circuit current of MDC-TENG at various gap distances. Test parameters: sliding distance: 10 cm, velocity: 1 m s^{-1}.

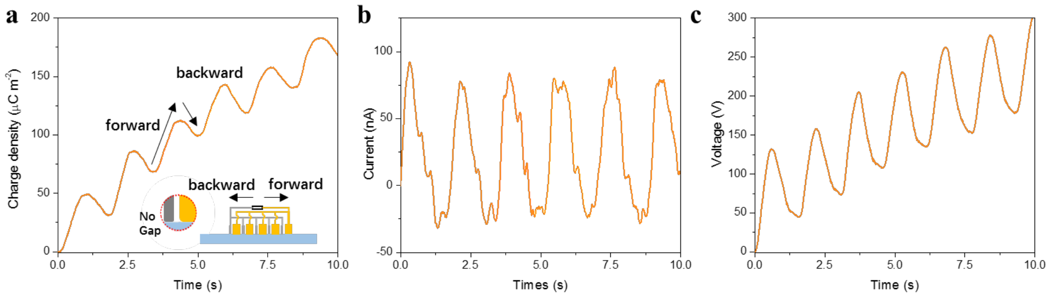

Supplementary Fig. 7. The output performance of MDC-TENG device with gap distance ~0 (MDC-TENG unit = 20, sliding distance = 10 cm, the inset is its schematic diagram): (**a**) charge density, (**b**) short-circuit current and (**c**) open-circuit voltage.

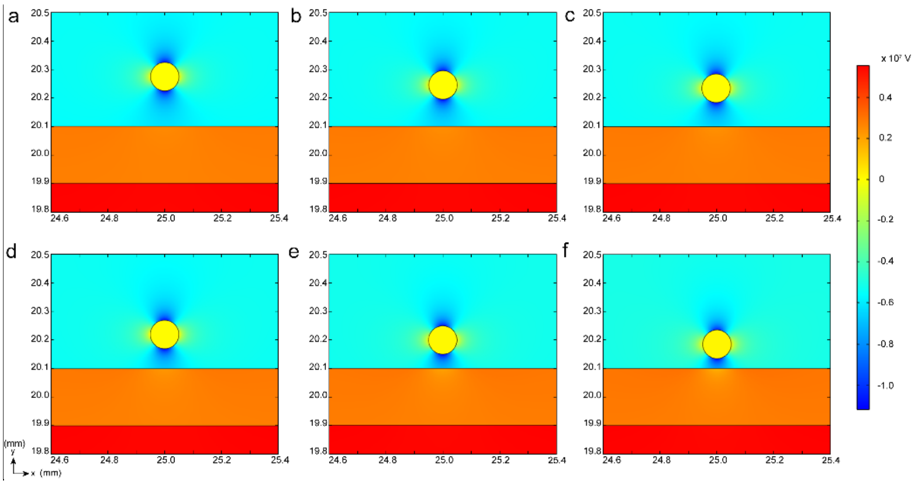

Supplementary Fig. 8. The electric field distribution of the charged PTFE film and the charge collecting electrode under different gap distances: (**a**) 125 μm, (**b**) 95 μm, (**c**) 85 μm, (**d**) 70 μm, (**e**) 50 μm, (**f**) 35 μm.

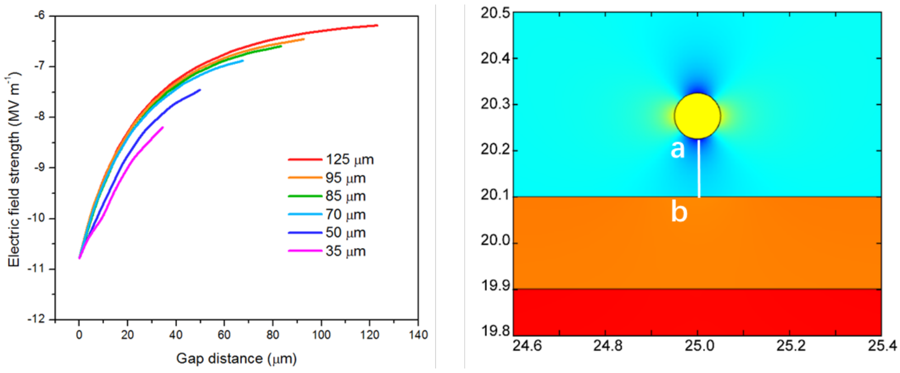

Supplementary Fig. 9. The electric field distribution from the charge collecting electrode (point a) to the charged PTFE film surface (point b) under different gap distances.

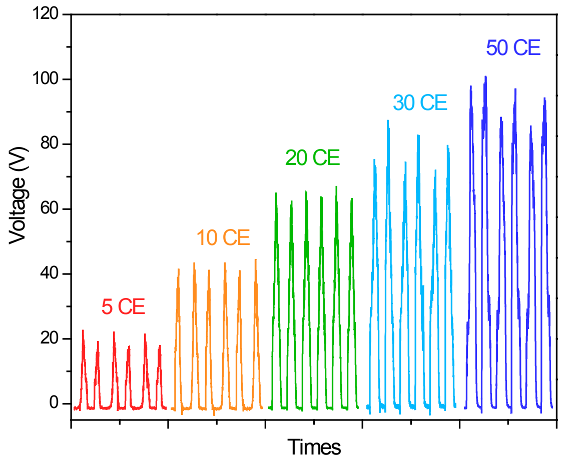

Supplementary Fig. 10. The open-circuit voltage of MDC-TENG with various MDC-TENG units. Test parameters: sliding distance: 10 cm, velocity: 1 m s^{-1}, friction layer: PTFE.

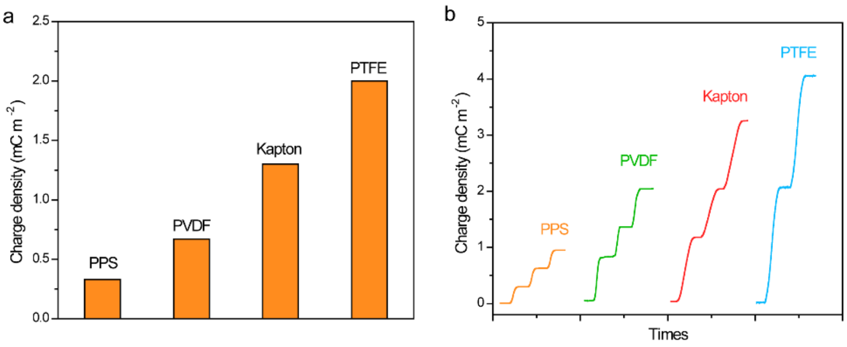

Supplementary Fig. 11. The charge density of MDC-TENG with various organic films as friction layer.

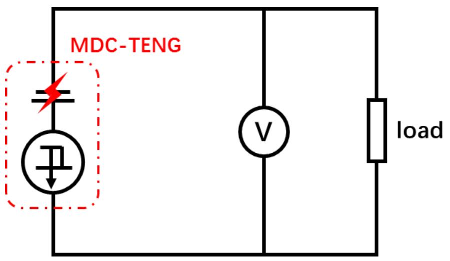

Supplementary Fig. 12. The circuit of MDC-TENG driving electronic devices directly (the external load is capacitors, LED bulbs or thermo-hygrometer).

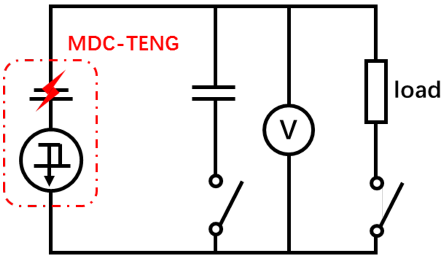

Supplementary Fig. 13. The circuit of MDC-TENG directly driving electronic devices with energy storage units.

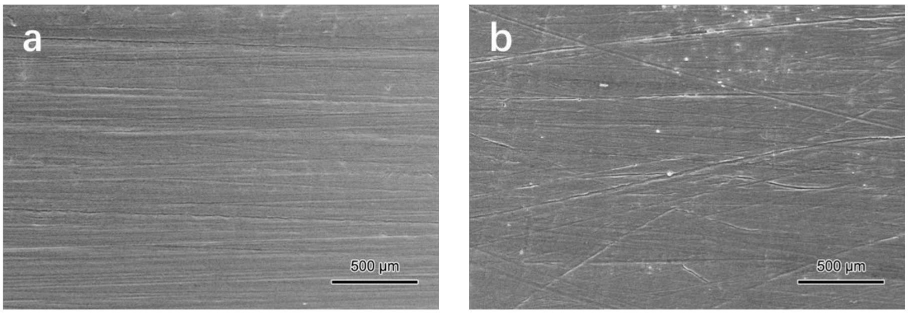

Supplementary Fig. 14. The SEM images of PTFE film surface: (**a**) before and (**b**) after 5000 cycles working test.

Supplementary Note 1: Working mechanism of DC-TENG

The fundamental mechanism of DC-TENG is on the basis of electrostatic breakdown. The schematic diagram of DC-TENG at different movement condition and their corresponding output current are shown in Supplementary Fig. 1. In the initial state (Supplementary Fig. 1a.), the left edge of friction electrode is coincident with PTFE's. Meanwhile, the friction electrode contacts with PTFE, resulting in the positive charges on the friction electrode and negative charges on the PTFE surface due to the contact electrification. Because of the electret nature feature of PTFE film, the negative charges are held and stayed on the PTFE surface for a long time. Thus, when the friction electrode starts to move forward, air breakdown will occur in the gap between the charge collecting electrode (positive charged) and PTFE surface (negative charged) because of a high electrostatic field inside the gap and a weak dielectric strength of air (3 kV mm^{-1}), resulting in the start of DC output peak (the poison point a in Supplementary Fig. 1d. As shown in Supplementary Fig. 1b and the inset schematic, during the friction electrode continues moving forward, the continuous air breakdown will make the electrons continuously flow from PTFE to the charge collecting electrode, releasing the contact electrification charges of the surface of the PTFE. Simultaneously, the collected charges flow back to the friction electrode through external circuit due to the potential difference, resulting in continuously direct current in the circuit (the poison point b in Supplementary Fig. 1d). The discharging process will stop when the right edge of friction electrode coincides with PTFE's (Supplementary Fig. 1c), at the same time, the DC output ends (the poison point c in Supplementary Fig. 1d). As discussed

above, the work mechanism of DC-TENG is the electrostatic breakdown process between charge collecting electrode and friction layer surface. The dielectric strength of friction dielectric film will not affect this process.

Supplementary Note 2: Development of charge density improvement for AC-TENG

The output performance of TENG is determined by its surface charge density quadratically [1]. Thus, how to improve the charge density is the crucial problem for the commercial applications of TENGs. According to the Equation (1), the surface charge density is limited by the $\sigma_{\text{triboelectrification}}$, $\sigma_{\text{r, air breakdown}}$, $\sigma_{\text{dielectric breakdown}}$. The optimization of friction materials and structure (e.g., soft contact) can enhance the $\sigma_{\text{triboelectrification}}$, thus, the charge density of AC-TENG increases from less than 0.05 mC m^{-2} to 0.25 mC m^{-2} [2, 3, 4]. However, the increased charge density met another limitation $\sigma_{\text{r, air breakdown}}$. The air breakdown occurred between friction electrode and friction layer surface will cause part of charges released. Many approaches were reported to address this bottleneck for optimizing the output performance of TENGs. High vacuum can avoid the air breakdown effect, and thus significantly improve the charge density of TENG to ~0.66 mC m^{-2} (PTFE/Cu). Meanwhile, combined with the spontaneous polarizations from ferroelectric material, the charge density can reach to ~1 mC m^{-2} (PTFE-BT/Cu) [5]. Whereas, TENGs always are operated in the air atmosphere for most applications. Thus, recently, on the basis of Paschen's law and TENG parallel-plate model, the ultrathin friction dielectric film is another strategy to elevate the threshold of $\sigma_{\text{r, air breakdown}}$. With the help of ion injection technique, the charge density of thin dielectric film TENG could be further improved to 1090 μC m^{-2} [6]. On the other hand, taking advantage of external circuit optimization, charge pump concept was carried out, which is devised to pump triboelectrification charges into the floating layer to achieve

accumulation and bind of charges (it avoids the limitation of $\sigma_{triboelectrification}$), and the charge density of AC-TENG can be improve to 1.02 mC m^{-2} in the air [7, 8]. By rational design of the voltage-multiplying circuits, the self-charge excitation TENG provided a high charge density ~1.25 mC m^{-2} as well as higher charge accumulation efficiency [9]. Very recently, combined with the charge-excitation and thin film (4 μm Polyetherimide film, PEI film) technique, an output charge density record of TENG was successfully increased to 2.38 mC m^{-2} in the air atmosphere [10].

Supplementary Note 3: Relationship between output charge and sliding distance

According to the mechanism of DC-TENG, during the sliding process, in unit distance d_x, the number of the output charges d_Q can be given as follow:

$$d_Q = \sigma_c W d_x \quad (1)$$

where the σ_c is the collected charge density from electrostatic breakdown, W is the width of slider. Thus, within a certain sliding distance l, the whole output charges Q can be written as follow:

$$Q = \int_0^l d_Q = \int_0^l \sigma_c W d_x = \sigma_c W l \quad (2)$$

As shown in Supplementary Equation (2) the output Q is linear to the sliding distance, confirmed by the experimental results in Fig. 3a,3b.

Supplementary Note 4: Relationship between I_{sc} and velocity

According to the definition, the current *I* is shown as follow:

$$I = \frac{d_Q}{d_t} \qquad (3)$$

where the *Q* is the output charges and *t* is the corresponding time.

Thus, bringing Supplementary Equation (2) into Supplementary Equation (3), because the σ_c and *W* is constant when the sliding speed is not intense (continuous high-speed motion will generate extra heat and rising temperature, which will affect the σ_c[11]), the Supplementary Equation (3) can be set as:

$$I = \sigma_c W \frac{d_l}{d_t} \qquad (4)$$

Because of the uniform motion of the slider, the $\frac{d_l}{d_t}$ is the velocity of slider. That is why the relationship is linear between short-circuit current and velocity.

Supplementary Note 5: Relationship between *dI/dt* and acceleration

For the uniform acceleration motion, the acceleration a is the second derivative of distance *l* to time *t*. Thus, derivative of time for both sides of Supplementary Equation (4), and it can be written as:

$$\frac{d_I}{d_t} = \sigma_c W \frac{d_l}{d_{t^2}} \tag{5}$$

Because the slider makes the uniform acceleration motion during the sliding process, according to the definition, the $\frac{d_l}{d_{t^2}}$ is the acceleration of slider. This explains the linear relationship between the $\frac{d_I}{d_t}$ and acceleration.

In the inset of Fig. 3f, the $\frac{d_I}{d_t}$ is set as the ratio of peak current value to its corresponding time which costs from 0 μA to peak value in one motion cycle.

Supplementary Note 6: Calculation of average strength of electrostatic field

The average electric field ($\bar{E}$) for the different gap distance between CCE and PTFE surface is calculated by integrating the respective electric field distribution curve $E(x)$ in Supplementary Fig. 6. The formula is shown as follow:

$$\bar{E} = \frac{\int_0^d E(x)d_x}{d} \tag{5}$$

where the *d* is the gap distance.

Supplementary References:

1. Zi, Y., Niu, S., Wang, J., Wen, Z., Tang, W. & Wang, Z.L. Standards and figure-of-merits for quantifying the performance of triboelectric nanogenerators. *Nat. Commun.* **6**, 8376 (2015).
2. Wang, J. *et al.* Sustainably powering wearable electronics solely by biomechanical energy. *Nat. Commun.* **7**, 12744 (2016).
3. Wang, S. *et al.* Maximum surface charge density for triboelectric nanogenerators achieved by ionized-air injection: methodology and theoretical understanding. *Adv. Mater.* **26**, 6720-6728 (2014).
4. Wang, Z., Cheng, L., Zheng, Y., Qin, Y. & Wang, Z.L. Enhancing the performance of triboelectric nanogenerator through prior-charge injection and its application on self-powered anticorrosion. *Nano Energy* **10**, 37-43 (2014).
5. Wang, J. *et al.* Achieving ultrahigh triboelectric charge density for efficient energy harvesting. *Nat. Commun.* **8**, 88 (2017).
6. Zhang, C. *et al.* Surface charge density of triboelectric nanogenerators: theoretical boundary and optimization methodology. *Appl. Mater. Today* **18**, 100496 (2020).
7. Cheng, L., Xu, Q., Zheng, Y., Jia, X. & Qin, Y. A self-improving triboelectric nanogenerator with improved charge density and increased charge accumulation speed. *Nat. Commun.* **9**, 3773 (2018).
8. Xu, L., Bu, T.Z., Yang, X.D., Zhang, C. & Wang, Z.L. Ultrahigh charge density realized by charge pumping at ambient conditions for triboelectric nanogenerators. *Nano Energy* **49**, 625-633 (2018).
9. Liu, W. *et al.* Integrated charge excitation triboelectric nanogenerator. *Nat. Commun.* **10**, 1426 (2019).
10. Liu, Y. *et al.* Quantifying contact status and the air-breakdown model of charge-excitation triboelectric nanogenerators to maximize charge density. *Nat. Commun.* **11**, 1599 (2020).
11. Liu D*, et al.* Hugely enhanced output power of direct-current triboelectric nanogenerators by using electrostatic breakdown effect. *Adv. Mater. Technol.* **5**, 2000289 (2020).
