# Supplementary material for: Rationally patterned electrode of direct-current triboelectric nanogenerators for ultrahigh effective surface charge density
Source: Nat Commun. 2020 Dec 3;11:6186. doi: 10.1038/s41467-020-20045-y (PMC7712892; doi:10.1038/s41467-020-20045-y)
Supplement: Supplementary file 3 — Description of Additional Supplementary Files [file 41467_2020_20045_MOESM3_ESM.pdf]

Description of Additional Supplementary Files

Supplementary Movie 1. Commercial LED bulbs were driven directly by MDC-TENG.

Supplementary Movie 2. Commercial thermo-hygrometer was driven directly by MDC-TENG.
